# Supplementary material for: Structure of a HIV-1 IN-Allosteric inhibitor complex at 2.93 Å resolution: Routes to inhibitor optimization
Source: PLoS Pathog. 2023 Mar 3;19(3):e1011097. doi: 10.1371/journal.ppat.1011097 (PMC10016701; doi:10.1371/journal.ppat.1011097)
Supplement: Attachment — Submitted filename: Cover_ReponseToReviewers_EilersGuptaetal.pdf [file ppat.1011097.s005.pdf]

Perelman School of Medicine
University of Pennsylvania

**Kushol Gupta, Ph.D.**
*Research Assistant Professor*

November 1st, 2022

Dear Madam or Sir,

Thank you very much for reviewing our manuscript, "*Structure of a HIV-1 IN-Allosteric Inhibitor Complex at 2.93 Å Resolution: Routes to Inhibitor Optimization*," by Eilers/Gupta et al. We are gratified that the three reviewers found merit in the paper, and we are very grateful for the suggestions for improvement. We have incorporated these suggestions as outlined below (highlighted using tracked changes in Microsoft Word):

**Reviewer 1.**

We are grateful to Reviewer #1 for recognizing the amount of work that went into producing the crystals needed for this study, and that the "*crystallographic work was carefully done and rigorously reported.*"

> *Reviewer #1: 1) The figures are beautiful, but it is a little confusing that the colors are inconsistent: sometimes the CTD is the white one and sometimes the CCD is the white one.*
>
> To help address this issue, we have updated Figure 4 so that the CCD remains in shades of blue throughout the manuscript.
>
> *2) Line 59 - (legend to Fig. 1) move "D" to next sentence.*
>
> Corrected.
>
> *3) Line 143 – define c(S) and c(M) for the non-specialist reader*
>
> Corrected – the passage now reads: "Fitting of data to the Lamm equation allows for the determination of a distribution of species, both in terms of sedimentation coefficient (c(S), and mass (c(M)). SV-AUC data collected at 20ºC with CCD•BI-224436•CTD(220-270)$^{L242A}$ allowed observation of discrete peak species in *c(S)* and *c(M)* distributions, confirming the formation of a ternary complex and higher-order species (S1D-E Fig)."
>
> *4) Line 189 - the "CCD is nearly symmetric" – "CCD dimer" would be clearer*
>
> Corrected – the passage now reads: "Although the CCD dimer is nearly symmetric in the ternary complex structure (Cα RMSD = 0.15 Å not including the α4 helices), the CTDs and the α4 helices adopt slightly different positions in the two halves of the complex."
>
> *5) Line 196 – should "protein accessible surface area" be "solvent-accessible protein surface area"?*
>
> Corrected – the passage now reads: "The ALLINI binding interface buries ~512 Å$^2$ of solvent-accessible protein surface area between the CTD and CCD dimer, and an additional 534 Å$^2$ (of 598 Å$^2$ total) of BI-224436 at each site."
>
> *6) Lines 201 (legend to Fig. 3) – should it be sigmaa-weighted (not sigma weighted) and in line 203 should it be "contoured at 1 sigma" not "weighted at 1 sigma"?*
>
> Corrected – the passage now reads: "B. Representative sigma A-weighted $2F_o$-$F_c$ electron density, contoured at 1σ at the first (left) and second (right) ALLINI binding sites."

242 Anatomy-Chemistry Building | Philadelphia, PA 19104 | 215.573-7260 | kgupta@upenn.edu

**Kushol Gupta, Ph.D.**
*Research Assistant Professor*

*7) Line 242 (legend to Fig. 5) – is this site I or site II?*

Amended to read: “A. Shown is a view of the site I BI-224436 binding site on the CCD, with the CTD omitted for clarity.”

*8) Line 267 – “Shows is a bound” should be “Shown is a bound”*

Spelling corrected.

*9) Line 324 – “resulting in xxx” – please finish thought.*

Corrected – the passage now reads “In serial passage resistance studies with BI-224436, the mutations A128T and A128N arise [37,58], which would be predicted to clash with the Tyr226 side-chain in addition to the R4 substituent noted earlier, resulting in a direct perturbation of CCD-CTD interface.”

*10) Line 329 – referring to figure S2 here rather than later would help the reader*

Amended as advised.

*11) Line 324 – specify ORDERED buried water molecules are rare?*

Done

*12) Lines 345-348: awkward and internally redundant sentence*

The passage has been rewritten for clarity: “This pocket was created by the observed conformational change in Trp-131 (S2 Fig) that occurs upon CTD binding. The density is best modelled by a molecule of ethylene glycol and is only observed in the more ordered ALLINI site 1 (Fig 6C).”

*13) Line 541 – the crystals were grown in batch mode rather than by the more usual vapor diffusion? Please clarify.*

Now clarified: “CCD, CTD, and BI-224436 were co-crystallized using hanging drop vapor diffusion by mixing 100 µM CCD, 100 µM CTD, and 500 µM BI-224436 in 20 mM Tris•HCL pH 7.4, 250-300 mM NaCl, and 1 mM DTT with 25% ethylene glycol.”

*14) Figure 5 – the lack of any contact between the inhibitor and W131 might be taken to undermine your model, but I’m guessing that W131 is important for CCD-CTD contacts? I’d recommend saying so in the text when you discuss this figure (those contacts do show up in Fig. 6, but don’t let the reader start thinking negative thoughts).*

A sentence has been added to that paragraph to further clarify: “While Trp-131 contributes significantly to the CCD-CTD protein interface stabilized by ALLINI binding, no specific drug interactions between Trp-131 and the drug occur.”

**Reviewer 2.**

We appreciate the reviewer’s view on the quality of our data and advances provided by our work.

Perelman School of Medicine
UNIVERSITY of PENNSYLVANIA

Kushol Gupta, Ph.D.
Research Assistant Professor

*The IN used here is actually two sub-domains (CCD and CTD), unlinked. I have some concerns as to whether these will form complexes that accurately reflect the way the intact IN might oligomerize. The overlap with earlier work (ref #10, PMID: 27935939) with full-length IN gives some comfort on this point. Perhaps some added comments on this would be helpful.*
*It remains somewhat uncertain whether the polymeric structures observed here are actually formed in vivo, but we probably have to accept this uncertainty.*

We provide an additional comment to address this concern:

(lines 174-177): “The structure of the *minimal* CCD•BI-224436•CTD ternary complex is shown in Fig 2. The overall quaternary arrangement surrounding the ALLINI resembles that observed in the structure of full-length IN•GSK1264, where the CTDs are connected by α-helical linkers to neighboring CCD dimers.”

(lines 219-221): “While it is known that CTD dimerization is necessary for drug-induced aggregation [52], it remains to be determined whether these observed CTD-CTD interfaces in this minimal structure exist with the full-length protein in virions.”

**Reviewer 3.**

We thank the reviewer for the very detailed comments and the important questions posed.

*Excitement is diminished by the fact that their work does not explain how other ALLINI scaffolds such as pyridine, isoquinoline, or indole-based scaffolds induce IN hyper-aggregation complexes. Is there observation only relevant for a select subgroup of ALLINIs highlighted by BI-224436 and GSK1264?*

*Can the authors speculate on the transformative nature of their work to other ALLINI scaffolds? If so, this would elevate the level of their findings and increase its appeal.*

*Reviewer #3: Major comments:*
*1- The authors need to address how other ALLINI types such as isoquinoline (Wilson et al, 2019), indole (Patel et al, 2016), thiophenecarboxylic acid (Patel et al, 2016), and other pyridines (Fader et al, 2016) “fit” within the ALLINI binding pocket of their structure (basically an expansion of Fig 7 and resulting discussion).*

*Is it only pyridine-based compounds that are tolerated? Or is this pocket more general to all ALLINIs except KF116 (a pyridine-based scaffold)? Such a conclusion would address the applicability of their work to the ALLINI field which is known to cover a wide diversity of chemical scaffolds. Such a study of the diversity would dramatically increase the appeal of their work to the broader audience.*

In this manuscript, we already extend our atomic model to a panel of prototype ALLINIs in Figure 7 to discuss the generalized features of ALLINI binding and predicted impact of distal ornaments on CTD interactions. We now further include a new supplemental figure (S3) along with revision of the results paragraphs that discuss these features to highlight the generality of this mode of binding to the isoquinoline, indole, and thiophenecarboxylic acid scaffolds. These scaffolds are also readily accommodated, based on available CCD-only crystal structures. Our modelling is limited to available crystallographic models of ALLINI-bound CCD.

*Is this because other ALLINIs such as KF116 (a pyridine-based scaffold) appears to induce tetramer-based aggregation whereas quinoline scaffolds can favor dimeric forms which could lead to the observed open polymers reported here?*

242 Anatomy-Chemistry Building | Philadelphia, PA 19104 | 215.573-7260 | kgupta@upenn.edu

**Kushol Gupta, Ph.D.**
*Research Assistant Professor*

ALLINI-induced aggregation is a complex phenomenon comprised of multiple specific interactions, including a) oligomerization of IN to first create the ALLINI binding site, b) the binding of ALLINI to the CCD dimer interface, c) the binding of CTD to the ALLINI-bound CCD, and d) the bridging CTD-CTD interactions that ensue within the branched polymer. Additionally, ALLINIs compete with LEDGF(IBD)-CCD interactions. How the diverse ALLINI scaffolds and their chemical ornaments affect each of these specific events is not yet entirely understood. The question posed by the reviewer (oligomer selection) is an excellent one, but outside of the scope of this study.

*2- On a related note, it would be nice to see an overlay of the structure of GSK1264 vs BI-224436 binding pockets to see if there are any key differences between the two structures solved by this group.*

An overlay between CCD-bound GSK1264 (4OJR), IN-GSK1264 (5HOT), and our BI-224436 complex is now provided in Figure S4, panel A.

However, a more direct comparison of our prior 4.4 A structure with GSK1264 with our new structure is limited by the disparity in resolution: at 4.4 A resolution, side chain positions could only be inferred and are heavily biased by higher resolution reference structures (using the DEN method), whereas in our work in this manuscript, we could directly model the side-chain electron density at higher resolution, with eight such binding sites in the asymmetric unit.

*3- The discovery that the two ALLINI binding sites appear to differ in the solved structure (in both "openness" and "order") is very intriguing. As the authors point out, it has been proposed that LEDGF/p75 could similarly have two different binding orientations that result in different binding affinities which appears to be the case for ALLINIs as well. Can the authors generate predicted binding affinities of these two pockets bound to BI-224436 using computational modeling? Do they exhibit a high and low affinity similar to LEDGF/p75? Is this what allows pyridine-based compounds to generate branched polymer chains that presumably consists of dimers and likely some tetramers? For these reasons, the reviewer would really like to see predicted binding affinities of these two sites!*

We agree that this is a very intriguing aspect of the solved structure. However, while a great idea, to calculate the differences in binding affinity *in silico* is non-trivial and would be a separate molecular dynamics study that lands well outside of our scope of expertise.

*4- It is unclear why the authors use a combination of CTD of 220-288 for DLS ,analytical ultracentrifugation, and aggregation assays but CTD 220-271 for crystallization. Did CTD 220-288 not work for crystallization? Does the last 17 aa make the complex unamenable for crystallization or was it just not tried? In reverse, what does CTD 220-288 behave like in the DLS or ultracentrifugation assays? One would like to see both used in at least one of the biophysical approaches to compare if the last 17 aa are playing a role in inhibitor induced aggregation or preventing the formation of higher order polymers.*

To clarify, we employed a monomeric L242A variant of CTD220-270 in our AUC experiments, similar to the wild-type truncation used in in our crystallization trials (220-271). In these crystallization trials, visibility turbidity is also apparent. This is now clarified in the text throughout.

We have not examined the contributions of these last 17 aa to aggregation and elected not to include them in our crystallization trials due to the detected disorder by SAXS (Gupta 2020). The last observed interacting CTD residues in our intact IN-GSK1264 structure (Gupta 2016) guided our construct selection.

Perelman
School of Medicine
UNIVERSITY of PENNSYLVANIA

**Kushol Gupta, Ph.D.**
*Research Assistant Professor*

*5- The authors provide a nice discussion of ALLINI resistance in the binding pocket and CTD. It would be interesting as too where Valine 165 lies within their open polymer and branched polymer structures. This mutation arises after selection under KF116 pressure and is predicted to affect CCD dimer interface to allow resistance (Hoyte 2017).*

We agree that this is a very interesting *ex vivo* resistance mutation against KF116. We have added the following sentence in our results (lines 296-301): "Notably, some *ex vivo* CCD mutations do not lie anywhere in proximity to the drug binding site or CCD-CTD interface but would be predicted to instead confer oligomeric defects to the enzyme, which is prerequisite to the formation of the ALLINI binding site. For example, A205P resides at the CCD dimer interface and renders resistance to both GSK002 and GSK1264, while the KF116 resistance mutation V165I confers oligomeric defects in IN."

*Reviewer #3: Minor comments:*
*1- Line 324 is incomplete.*

Corrected – the passage now reads "In serial passage resistance studies with BI-224436, the mutations A128T and A128N arise, which would be predicted to clash with the Tyr226 side-chain in addition to the R4 substituent noted earlier, resulting in a direct perturbation of the CCD-CTD interface."

Throughout the revision, we also made minor corrections and revisions for the sake of clarity and improvement.

We thank the reviewers and editor for their helpful comments, and hope that the revised manuscript is now suitable for publication in PLoS Pathogens.

Sincerely,

Kushol Gupta

Frederic Bushman

Greg Van Duyne

242 Anatomy-Chemistry Building | Philadelphia, PA 19104 | 215.573-7260 | kgupta@upenn.edu
